# The Trends in Excess Mortality in Winter vs. Summer in a Sub-Tropical City and Its Association with Extreme Climate Conditions

**DOI:** 10.1371/journal.pone.0126774

**Published:** 2015-05-20

**Authors:** Pui Hing Chau, Jean Woo

**Affiliations:** 1 School of Nursing, The University of Hong Kong, Hong Kong; 2 Department of Medicine and Therapeutics, The Chinese University of Hong Kong, Hong Kong; University of Vigo, SPAIN

## Abstract

While there is literature on excess winter mortality, there are few studies examining the evolution of its trend which may be changing in parallel with global warming. This study aimed to examine the trend in the excess mortality in winter as compared to summer among the older population in a sub-tropical city and to explore its association with extreme weather. We used a retrospective study based on the registered deaths among the older population in Hong Kong during 1976-2010. An Excess Mortality for Winter versus Summer (EMWS) Index was used to quantify the excess number of deaths in winter compared to summer. Multiple linear regressions were used to analyze the trends and its association with extreme weather. Overall, the EMWS Index for ischemic heart disease, cerebrovascular diseases, chronic lower respiratory diseases, pneumonia, and other causes were 43.0%, 34.2%, 42.7%, 23.4% and 17.6%, respectively. Significant decline was observed in the EMWS Index for chronic lower respiratory diseases and other causes. The trend in the index for cerebrovascular diseases depended on the age group, with older groups showing a decline but younger groups not showing any trend. Meteorological variables, in terms of extreme weather, were associated with the trends in the EMWS Index. We concluded that shrinking excess winter mortality from cerebrovascular diseases and chronic lower respiratory diseases was found in a sub-tropical city. These trends were associated with extreme weather, which coincided with global warming.

## Introduction

Environmental temperature is known to aggravate or precipitate mortality. Higher mortality rates were reported on both hot and cold days [1–4]. Cardiovascular diseases and respiratory diseases are more sensitive to the extreme weather, both in the Western populations [1,5–7] and the Chinese populations [4,8]. Furthermore, the older population is more vulnerable to the influence of temperature [1,9–13].

While temperature has been shown to be strongly associated with mortality, other meteorological variables like relative humidity and atmospheric pressure also play a role although the effects were inconsistent [1,13–16]. Some researchers used indices to summarize the combined effect from various meteorological variables and examined their relationship with mortality [16,17]. One of such indices was net effective temperature (NET), which was a single measure to summarize the combined effect of air temperature, relative humidity and wind speed on human feeling of thermal comfort [18].

Located in the sub-tropical region, Hong Kong has hot and humid summers, and cold and dry winters. According to the Hong Kong Observatory, due to global warming and local urbanization, there was an average rise in mean temperature of 0.15°C per decade in 1947–2013 and an increase of 0.20°C per decade in 1984–2013; and extremely cold days have become rarer whilst extremely hot days become more frequent [19]. In Hong Kong, excess deaths were associated both high and low temperatures, but the effect of cold weather appeared to be more obvious. Circulatory and respiratory related deaths were shown to associate with the daily minimum NET in winter but not the daily maximum NET in summer [11]. Using a Weather Stress Index (WSI), a relationship between low WSI and mortality in winter was shown, but no conclusive findings for summer [20]. Yan confirmed a winter peak in all-causes, circulatory and respiratory related mortality among the older age groups [15]. A study conducted in Brisbane, an Australian city in the sub-tropical region also reported higher all-causes mortality in winter than in summer [9]. A Chinese city in the sub-tropical region—Guangzhou showed higher mortality in winter than non-winter [13]. These findings suggest that the older population in a subtropical region is likely to be affected by cold winter more than the hot summer.

There is growing concern over global warming. It is projected that at the end of 21^st^ century, the global average surface temperature is likely to increase by 0.3–4.8°C as compared to the average of 1986–2005 [21]. Some researchers predicted a reduction in cold-related deaths with an increase in heat-related deaths [22,23], but the increases in heat-related deaths could not be compensated by decreases in cold-related deaths [24–26]. In such scenario, the relative adverse effect of cold winter versus hot summer might be reducing. Despite of the well-known J-shaped relationship between mortality and temperature, few studies in the sub-tropical region investigated how this relationship evolved in the past decades. A recent Korean study reported varying effects of temperature on mortality across time and causes of death [27].

The objectives of this study were (i) to examine the trend in the excess mortality in winter as compared to summer among the older population in a sub-tropical city; and (ii) explore the association between extreme weather observations and such excess mortality.

## Methods

### Data collection

In Hong Kong, all natural deaths attended by registered medical practitioner were registered at the Births and Deaths Registry. The medical practitioner attended the deceased would certify the causes of death. All unnatural deaths were investigated by the Coroner system such that all deaths had to satisfy the “beyond reasonable doubt” criterion. The causes of death were then determined by the Coroner and registered at the Births and Deaths Registry. The Census and Statistics Department (C&SD) will then compile the statistics, with the Department of Health assisting in the validation process. Details had been described earlier [28].

The C&SD keeps two series of death files, namely “known deaths” and “registered deaths”. The known death file of a particular year includes all deaths that occurred in that year and registered before the deadline. The known deaths file is affected by the reporting delay, especially for deaths occurring in the last few months of the year and the unnatural deaths which have longer delay [29]. On the contrary, the registered death file of a particular year includes deaths registered in that year, covering deaths occurred in that year or before. Usually registered death files of a particular year and the subsequent two years are needed so as to retrieve all deaths occurred in a particular year. In this study, registered death files for the years 1976 (the earliest available year) to 2012 were used. Hence, the data series were valid for analysis of deaths up to early 2011.

The deaths were classified into summer and winter according to the month of death. In a particular year, summer was defined as from June to August; and winter, December of the year to February of the next year. As we aimed to contrast the number of death in winter (the coldest months) and summer (the hottest months), deaths in spring and autumn were not used in the analysis. Since older people are more vulnerable to the weather [9,11,13,15], the older population aged 65 years and above was studied. Three subgroups were classified according to age (65–74, 75–84, ≥85).

The causes of death were identified by International Classification of Diseases (ICD). Different versions have been used for different years by the C&SD and explanatory notes were given in the user guide. Since cardiovascular and respiratory deaths are more sensitive to extreme weather [1,5–8], four related causes were extracted for subgroup analysis, namely ischemic heart disease, cerebrovascular diseases, chronic lower respiratory diseases and pneumonia. Causes other than these were grouped as others. Although influenza and falls may be sensitive to season [30,31], it was not extracted for subgroup analysis due to the small number of deaths from these causes. S1 Appendix shows the ICD codes for the specific causes of death.

Meteorological data from 1976 to 2010 were obtained from the Hong Kong Observatory to construct extreme weather variables, which were selected based on literature about their adverse effect on mortality. Ambient temperature had been shown to associate with mortality among the older population [4,8,14]. Hence, the number of cold days (days with daily minimum temperature ≤12°C) in winter, the number of very hot days (days with daily maximum temperature ≥33°C) in summer and the number of hot nights (days with daily minimum temperature ≥28°C) in summer which were defined on the ambient temperature were extracted. Since the feeling of discomfort depends on ambient temperature, relative humidity and wind speed, Leung and coworkers [11] had shown that NET above 26°C or below 14°C was likely to increase mortality rates of the local older population. We calculated the daily NET based on the standard formula [18], and counted the numbers of days with NET above 26°C in summer and the number of days with NET below 14°C in winter. Previous studies showed that larger daily temperature range (calculated by subtracting daily minimum temperature from daily maximum) was related to higher mortality among the Chinese population [32–34]. In Hong Kong, only 5% of the days had a daily temperature range over 6.8°C in the past 35 years. Therefore, we counted the number of days with daily temperature range over 6.8°C in summer and in winter respectively. The effects of relative humidity on local mortality were inconsistent. One study suggested that lower relative humidity was associated with higher mortality in winter [14], but an earlier study could not found a significant association [15]. In Hong Kong, about 5% of the days had a relative humidity over 93% and about 5% of the days had a relative humidity of below 58% in the past 35 years. Thus, we counted the number of days with relative humidity over 93% in summer and those below 58% in winter. Furthermore, while a Guangzhou study atmospheric pressure was not significantly correlated with excess winter mortality [13], a study in Spain suggested higher mortality was related to higher air pressure [35]. In the past 35 years in Hong Kong, about 5% of the days had an atmospheric pressure over 1023.4hPa, and 5% of days below 1002.9hPa. Hence, we counted the number of days with atmospheric pressure over 1023.4hPa in winter and the number of days with atmospheric pressure below 1002.9hPa in summer.

### Statistical analysis

To quantify the excess of deaths in winter as compared to summer, an index named Excess Mortality in Winter versus Summer (EMWS) Index was developed in a similar manner as the Excess Hospitalization in Winter versus Summer Index which had been developed to quantify the excess winter hospitalization [36]. These indices were largely modelled on the commonly used Excess Winter Mortality Index [13,37–39]. The EMWS Index, expressed in percentage points, was defined by:

$$EMWS\;Index=\;\frac{n_{w}-\left(n_{s}\times \frac{K_{w}}{K_{s}}\right)}{n_{s}\times \frac{K_{w}}{K_{s}}}\times 100\%$$

where n_w_ and n_s_ are the number of deaths in winter and summer respectively, and K_w_ and K_s_ are the number of calendar days in winter and summer respectively. Since the numbers of days in summer and winter differ, the number of deaths in summer was multiplied with the ratio (K_w_/K_s_) to give the expected number of deaths in summer assuming the number of days was the same as in winter. EMWS Index was expressed as a percentage change in the observed number of deaths in winter as compared with the adjusted number of deaths in summer. A positive index indicated that there was higher mortality in winter compared to those in summer after adjusting for the number of calendar days in the two seasons, and vice versa. The larger the index, the greater the excess in mortality in winter. Unlike the usual mortality rate which has to be adjusted by the population size, this index does not require adjustment for the population size since it is a ratio. EMWS Index breakdown by gender, age group and causes of death were compiled from summer of 1976 to winter of 2010 (which extends to February 2011).

First, multiple linear regressions were used to assess the temporal trend in the EMWS Index for each of the causes of death, with age group, gender, and year as independent variables. To investigate if the trends were different in the age groups and gender, the interactions of these variables with year were included as independent variables in the initial model. Insignificant interactions would be removed to construct the final model.

Second, multiple linear regressions were used to examine the association between the EMWS Index and the extreme weather in summer and winter of the corresponding years. The extreme weather variables in winter included number of cold days, number of days with NET below 14°C, number of days with the daily temperature range exceeding 6.8°C, the number of days with relative humidity below 58%, and the number of days with atmospheric pressure over 1023.4hPa. The extreme weather variables in summer included number of very hot days, number of hot nights, number of days with NET above 26°C, number of days with the daily temperature range exceeding 6.8°C, the number of days with relative humidity above 93%, and the number of days with atmospheric pressure below 1002.9hPa. Separate models were used to examine the EMWS Indices for different causes of death, since the indices might be related to different extreme weather variables. In each model, the EMWS Index was the dependent variable, age group and gender were included as independent variables in order to control for age group and gender effects. The extreme weather variables in winter and summer were selected to the model by stepwise procedure. Finally, calendar year was added to the models to examine if trends in the index existed after controlling for the significant extreme weather variables. Multicollinearity among the predictors in the final model was assessed by variance inflation factor (VIF). A VIF of less than 5 were considered as acceptable. Simple linear regression was used to analyze the trend in each of the extreme weather variables.

SPSS version 20.0 was used for the analysis. A significance level of 5% was adopted for the hypothesis testing and 95% confidence intervals (CI) were constructed for the regression coefficients.

### Ethics statement

Ethics approval was not required for the analysis of death data.

## Results

**Fig 1:**
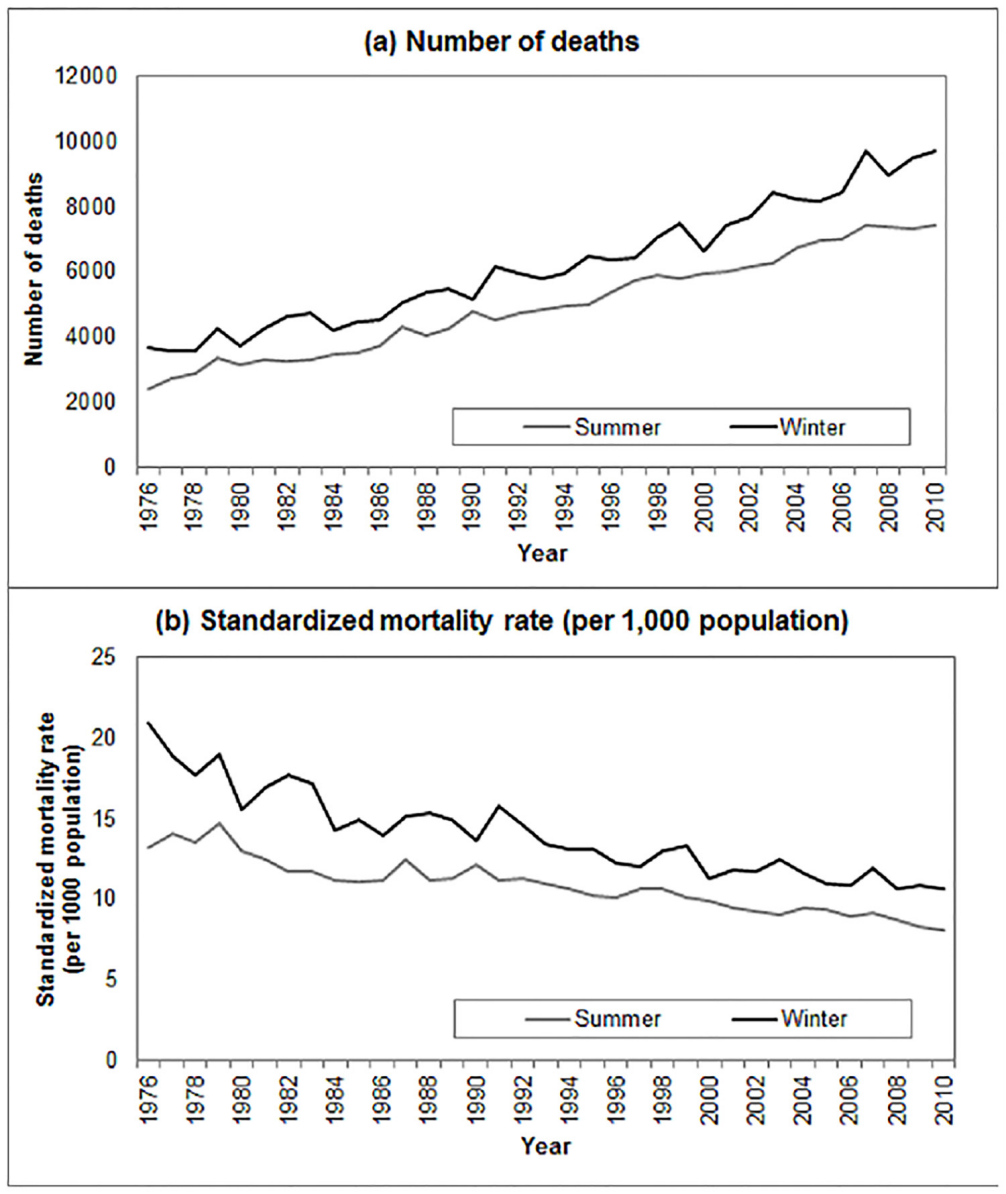
Mortality trends among the older Hong Kong population, 1976–2010. (A) Number of deaths. (B) Age-standardized mortality rate (per 1,000 population).

The registered death files of 1976 to 2012 recorded 1,170,722 deaths, of which 1,710 (0.1%) had missing year of death, month of death, age and/or gender. From 1976’s spring to 2010’s winter, a total of 755,109 deaths occurred among the Hong Kong population aged 65 and above, of which 173,607 (23.0%) occurred in summer, and 217,065 (28.7%) occurred in winter. Among the 390,672 deaths occurring in summer and winter, 46,677 (11.9%) were from ischemic heart disease, 45,793 (11.7%) were from cerebrovascular disease, 29,977 (7.7%) were from chronic lower respiratory diseases and 47,729 (12.2%) were from pneumonia. The number of deaths occurring in winter increased from 3,660 in 1976 to 9,726 in 2010 (a 2.7-fold increase), and those occurred in summer increased from 2,392 to 7,443 over the same period (a 3.1-fold increase). In 1976, the number of deaths in winter exceeded that in summer by 1,268 deaths, equivalent to 53.0% of deaths in summer; and in 2010, although such excess increased to 2,283 deaths, the excess in terms of percentage shrank to 30.7% only. The age-standardized mortality rate (per 1,000 population) in winter decreased from 20.87 in 1976 to 10.59 in 2010 (decreased by 49.3%), while that in summer decreased with a slower rate, from 13.23 in 1976 to 8.10 in 2010 (decreased by 38.8%). (Fig 1)

**Fig 2:**
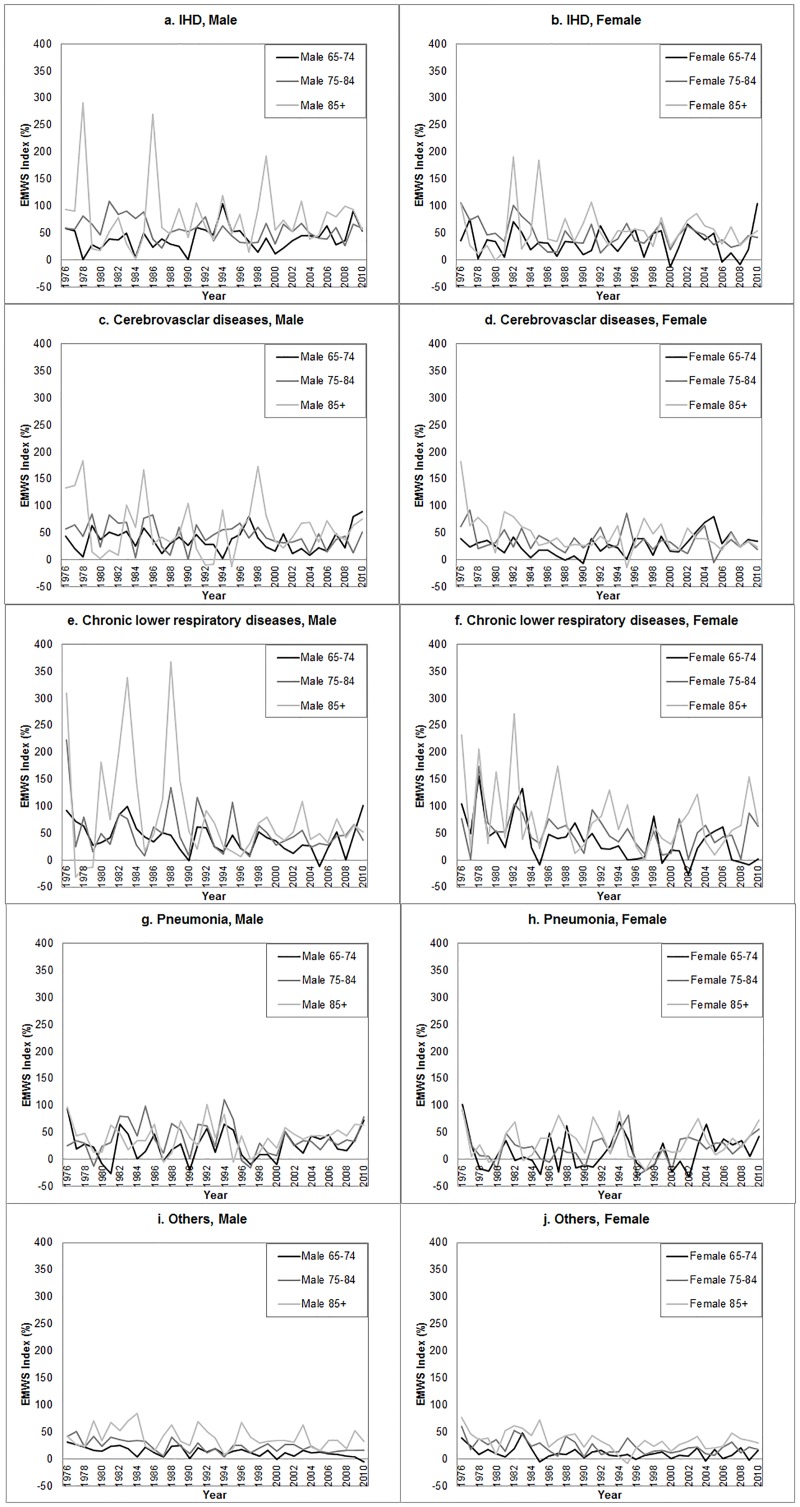
Trends in EMWS Index by causes of death, gender and age group, 1976–2010. (A) IHD, Male. (B) IHD, Female. (C) Cerebrovascular diseases, Male. (D) Cerebrovascular diseases, Female. (E) Chronic lower respiratory diseases, Male. (F) Chronic lower respiratory diseases, Female. (G) Pneumonia, Male. (H) Pneumonia, Female. (I) Others, Male. (J) Others, Female.

On average, the EMWS Index for ischemic heart disease, cerebrovascular diseases, chronic lower respiratory diseases, pneumonia, and other causes were 43.0%, 34.2%, 42.7%, 23.4% and 17.6% respectively. Multiple linear regressions showed that younger age group had significantly lower EMWS Index than the older age groups regardless of the causes of death. Furthermore, men had significantly higher EMWS Index than women for ischemic heart disease, cerebrovascular diseases and pneumonia, but not significantly different for chronic lower respiratory diseases and other causes. Fig 2 shows the trends in EMWS Index by gender, age group and causes of death. Multiple linear regressions showed the trends in the EMWS Index varied across causes of death. The EMWS Index significantly decreased for chronic lower respiratory diseases and other causes, regardless of gender or age groups. However, the trends in EMWS Index for cerebrovascular diseases varied across age groups, with the older population aged 75 or above experienced a significant decline, but the younger population aged 65–74 did not show any trend. Meanwhile, no significant trend was observed for the EMWS Index of ischemic heart disease and pneumonia. Table 1 shows the summary statistics of the EMWS Index by different gender, age groups and causes of death, as well as the fitted trends in the index across the years. (Fig 2)

**Table 1:** Summary statistics of EMWS Indices (in percentages) among Hong Kong population aged 65 and above, 1976–2010.

Causes of Death	Gender	Age	Mean	Median	Standard Deviation	Interquartile Range	Minimum	Maximum	Trend#
Ischemic heart disease	Male	65–74	40.29	39.83	22.23	27.85	0.94	104.44	NS
		75–84	57.03	57.02	20.34	28.13	21.98	108.53	
		≥85	80.27	62.63	62.03	46.79	2.22	291.85	
	Female	65–74	33.25	34.24	25.97	38.48	-12.38	104.44	
		75–84	47.50	45.63	23.37	36.27	13.93	106.41	
		≥85	56.09	51.74	41.16	40.25	-0.78	190.94	
Cerebrovascular diseases	Male	65–74	36.60	37.41	21.17	26.31	2.22	89.01	Aged 65–74:
		75–84	45.42	43.11	23.31	34.09	1.32	84.36	NS
		≥85	60.64	47.25	51.22	59.26	-12.94	183.95	Aged 75–84:
	Female	65–74	27.76	25.74	19.04	25.11	-6.82	80.67	-0.7 per year
		75–84	35.39	31.77	20.49	22.58	-5.30	92.42	Aged ≥85:
		≥85	45.66	38.58	32.75	34.95	-14.02	182.61	-1.2 per year
Chronic lower respiratory	Male	65–74	40.82	40.96	27.46	35.98	-11.72	100.59	-1.7 per year
diseases		75–84	52.10	43.79	42.55	40.55	6.42	222.39	
		≥85	84.23	52.59	94.62	77.09	-31.85	367.30	
	Female	65–74	36.84	26.90	40.39	51.11	-27.59	155.56	
		75–84	52.17	51.78	34.72	38.17	2.22	174.72	
		≥85	79.39	64.60	65.77	71.49	0.14	271.72	
Pneumonia	Male	65–74	26.11	22.32	27.45	38.01	-25.56	93.68	NS
		75–84	38.66	33.72	29.68	38.72	-15.28	110.46	
		≥85	41.82	43.11	25.87	38.68	-5.03	101.44	
	Female	65–74	13.71	5.87	31.89	49.53	-31.85	101.04	
		75–84	21.55	21.87	27.20	32.86	-30.35	91.46	
		≥85	32.80	27.78	29.86	38.33	-19.59	93.68	
Others	Male	65–74	13.98	13.36	8.56	14.59	-4.50	32.01	-0.6 per year
		75–84	24.15	24.40	11.38	17.83	4.38	51.64	
		≥85	40.69	34.76	19.16	26.57	9.57	84.97	
	Female	65–74	11.69	9.34	10.90	11.93	-4.72	47.88	
		75–84	23.17	21.18	13.44	18.03	5.02	60.44	
		≥85	34.82	34.11	17.73	21.84	-7.86	77.13	

Note:

^#^ Models without adjusting for meteorological variables;

NS: Not significant

**Fig 3:**
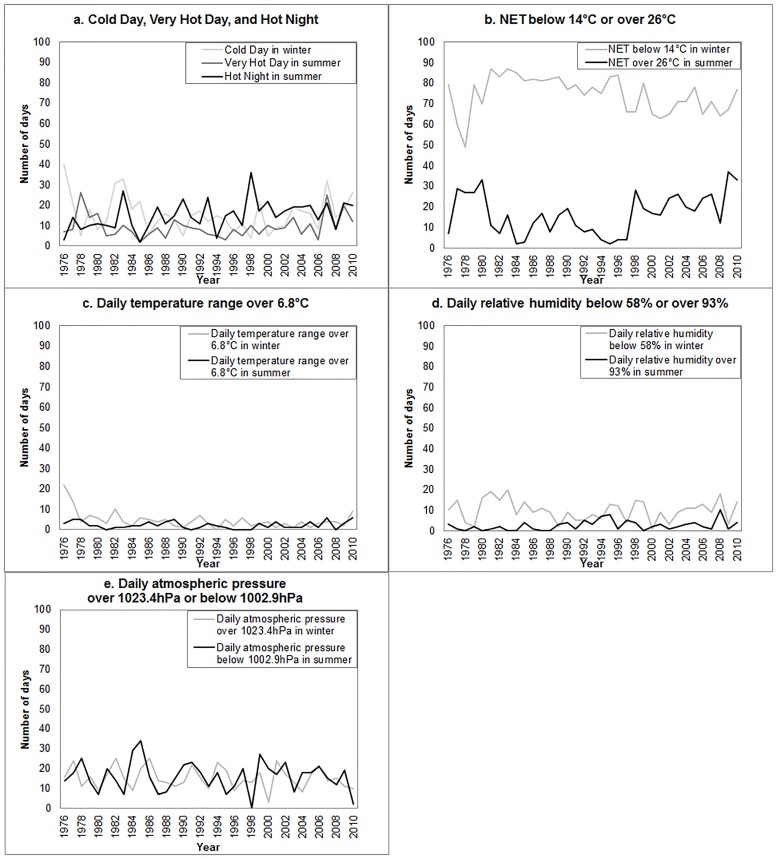
Trends in the number of days with extreme weather conditions in Hong Kong, 1976–2010. (A) Cold Day, Very Hot Day, and Hot Night. (B) NET below 14°C or over 26°C. (C) Daily temperature range over 6.8°C. (D) Daily relative humidity below 58% or over 93%. (E) Daily atmospheric pressure over 1023.4hPa or below 1002.9hPa.

Table 2 shows the summary statistics of the annual extreme weather variables used in the regression analysis. In winter, there were on average 15.7 days with daily minimum temperature below 12°C (Cold Days), 74.5 days with daily NET below 14°C in winter, 4.7 days with daily temperature range over 6.8°C, 9.9 days with daily relative humidity below 58%, and 15.3 days with daily atmospheric pressure over 1023.4hPa. In summer, there were on average 9.4 days with maximum temperature above 33°C (Very Hot Days), 15.0 days with minimum temperature above 28°C (Hot Nights), 16.5 days with daily NET above 26°C in winter, 2.2 days with daily temperature range over 6.8°C, 2.5 days with daily relative humidity over 93%, and days 15.9 days with daily atmospheric pressure below 1002.9hPa. The weather in winter became less stressful over the past 35 years that the number of winter days with large daily temperature fluctuation (daily temperature range over 6.8°C) decreased by 0.2 days per year and the number of winter days with high atmospheric pressure (daily atmospheric pressure over 1023.4hPa) decreased by 0.1 days per year. However, summers became more stressful as the number of Hot Nights in summer increased by 0.3 days per year and the number of summer days with very high humidity (daily relative humidity over 93%) increased by 0.1 days per year. Other extreme weather variables did not show significant trends. Fig 3 shows the trends in the meteorological variables over the study period. (Fig 3)

**Table 2:** Summary statistics of annual extreme weather variables in summer and winter, Hong Kong, 1976–2010.

Season	Extreme Weather Variables	Mean	Median	Standard Deviation	Interquartile Range	Minimum	Maximum	Trend
Winter	Number of Cold Days	15.7	15.0	8.7	10.0	4.0	40.0	NS
	Number of days with daily NET below 14°C	74.5	77.0	8.9	16.0	49.0	87.0	NS
	Number of days with daily temperature range over 6.8°C	4.7	4.0	4.1	4.0	0.0	22.0	-0.2 days per year
	Number of days with daily relative humidity below 58%	9.9	9.0	5.1	9.0	1.0	20.0	NS
	Number of days with daily atmospheric pressure over 1023.4hPa	15.3	15.0	5.3	8.0	3.0	25.0	-0.1 days per year
Summer	Number of Very Hot Days	9.4	8.0	5.5	5.0	2.0	26.0	NS
	Number of Hot Nights	15.0	14.0	7.1	10.0	2.0	36.0	+0.3 days per year
	Number of days with NET over 26°C	16.5	16.0	9.9	18.0	2.0	37.0	NS
	Number of days with daily temperature range over 6.8°C	2.2	2.0	1.8	3.0	0.0	6.0	NS
	Number of days with daily relative humidity over 93%	2.5	2.0	2.4	3.0	0.0	10.0	+0.1 days per year
	Number of days with daily atmospheric pressure below 1002.9hPa	15.9	17.0	7.4	9.0	0.0	34.0	NS

Note: Cold Days: days with minimum temperature below 12°C; Very Hot Days: days with maximum temperature over 33°C; Hot Nights: days with minimum temperature over 28°C; NET: Net Effective Temperature; NS: Not significant

Table 3 shows the fitted coefficients of final models of multiple linear regressions of the EMWS Indices. Controlling for the effects from gender and age group, three of the extreme weather variables in winter and five of the extreme weather variables in summer were selected by stepwise procedure to be predictors to the EMWS Indices for different causes of death. For all causes of death, a more stressful winter, as reflected by more days with extremely low temperature (Cold Days), more days with high atmospheric pressure (daily atmospheric pressure over 1023.4hPa), and more days with larger daily temperature fluctuation (daily temperature range over 6.8°C), was associated with a higher EMWS Index. On the other hand, more stressful summer, as reflected by more days with uncomfortably hot weather (daily NET over 26°C), more days with high humidity (daily relative humidity over 93%), and more days with low atmospheric pressure (daily atmospheric pressure below 1002.9hPa), was generally associated with smaller EMWS Index. Nevertheless, for pneumonia, stressful summer weather conditions were associated with higher EMWS Index, rather than a lower index.

**Table 3:** Fitted regression coefficients of final models of multiple linear regressions of the EMWS Indices.

Factors	Ischemic Heart Disease		Cerebrovascular Diseases		Chronic Lower Respiratory Diseases		Pneumonia		Others	
***Factors included intentionally***	Fitted Regression Coefficients (95% Confidence Intervals)										
Intercept	30.02	(8.95,51.09)	41.00	(27.32,54.67)	123.59	(89.87,157.31)	-16.43	(-32.05,-0.81)	38.23	(32.16,44.30)
Gender										
Male	13.58	(4.24,22.92)	11.28	(3.40,19.16)	2.92	(-10.28,16.11)	12.84	(6.02,19.66)	3.05	(-0.04,6.13)
Female (reference)	0		0		0		0		0	
Age group										
65–74	-31.41	(-42.85,-19.97)	-20.98	(-30.63,-11.32)	-42.98	(-59.14,-26.82)	-17.40	(-25.75,-9.04)	-24.92	(-28.70,-21.14)
75–84	-15.91	(-27.35,-4.48)	-12.75	(-22.40,-3.10)	-29.68	(-45.84,-13.51)	-7.20	(-15.56,1.15)	-14.10	(-17.88,-10.32)
85 and above (reference)	0		0		0		0		0	
***Factors selected by stepwise procedure***										
***Winter variables***										
Number of Cold Days	0.75	(0.19,1.30)	—		—		1.12	(0.70,1.54)	0.68	(0.44,0.92)
Number of days with daily atmospheric pressure over 1023.4hPa	1.40	(0.50,2.30)	—		—		1.06	(0.38,1.74)	—	
Number of days with daily temperature range over 6.8°C	—		1.92	(0.83,3.01)	3.64	(1.72,5.56)	—		0.05	(-0.52,0.62) ^#^
***Summer variables***										
Number of Very Hot Days	—		—		2.30	(0.64,3.96)	—		—	
Number of days with NET over 26°C	—		—		-1.66	(-2.70,-0.61)	—		-0.06	(-0.26,0.14)^#^
Number of days with daily relative humidity over 93%	—		—		-5.93	(-9.44,-2.42)	2.30	(0.67,3.94)	-1.25	(-2.08,-0.42)
Number of days with daily temperature range over 6.8°C	—		—		—		2.34	(0.29,4.38)	-1.04	(-2.02,-0.06)
Number of days with daily atmospheric pressure below 1002.9hPa	—		—		-2.01	(-2.96,-1.06)	—		—	
***Trend after controlling demographical and extreme weather factors***										
Year^**§**^	-0.09	(-0.57,0.38)	-0.13	(-0.57,0.30)	-0.43	(-1.32,0.47)	0.15	(-0.23,0.52)	-0.37	(-0.58,-0.15)

Note: The unit of the fitted coefficients and the 95% CI are percentage points; Only significant extreme weather variables were selected to the final models by stepwise procedure;

- denotes insignificant factors to the EMWS Index for that cause of death;

^**§**^ Year = 1 denotes the year 1976, year = 2 denotes the year 1977, and so on;

^#^ These variables became insignificant when calendar year was included in the model;

Multicollinearity was not detected in the final models.

After controlling for the extreme weather variables selected by the stepwise procedure, calendar year was found to be insignificant in predicting the EMWS Index for the four selected causes of mortality. However, a significant declining trend remained in other causes of mortality and two extreme weather variables became insignificant because of the inclusion of calendar year as covariate.

## Discussion

Using a time series of mortality data spanning over three decades, this study quantified the relative effect of winter on mortality as compared to summer among the older population in a city located in a sub-tropical region—Hong Kong through the calculation of the EMWS Index. This study investigated the trends in such index to explore if the relative effect of winter versus summer has been evolving. Significant declining trends were found in the EMWS Index for cerebrovascular diseases, chronic lower respiratory diseases and other causes, with exception of those aged 65–74 not showing any trend of EMWS Index of cerebrovascular diseases. The Index for chronic lower respiratory diseases experienced faster decline than the others. Extreme weather variables, which coincided with global warming, could largely explain the declining trends in the EMWS Index for cerebrovascular diseases and chronic lower respiratory diseases. However, the declining trend in the EMWS Index for other causes of death could not be explained by extreme weather variables.

Our findings showed that the EMWS Index of chronic lower respiratory diseases and cerebrovascular diseases experienced a decline, and such declining trend could be largely captured by the trends in the extreme weather variables. A decreasing EMWS Index indicated shrinking excess winter mortality, that is, narrowing of the differences between the effects from hot and cold weather. Since a declining trend was observed for the age-standardized mortality rate in both winter and summer, the shrinking of excess winter mortality was associated with the a slower declining rate of mortality in summer than in winter, rather than an actual increase in the summer mortality rate. Hence, Hong Kong has not observed an increase in heat-related deaths as predicted in the Western literature [22,23]. In Hong Kong, the past experiences showed the effect of hot weather was less prominent as compared to cold weather [36,40]. Specific programs have been developed to reduce the winter surge in the usage of healthcare services [41]. If the shrinking trend continues, the healthcare burden resulted from the winter surge may be alleviated in long term, while some of the resources may be diverted to address the increased healthcare demand in summer. Nevertheless, since such an evolution process is likely to be slow, the surge in healthcare demand during winter surge will likely continue for a substantial period.

The trend in the EMWS Index of cerebrovascular diseases varied across age groups, with only the older age groups showing significant decreasing trend. The absence of decreasing trend in the age group 65–74 may be expected since the association between hospital admissions for ischemic stroke and temperature was stronger in older age groups [42]. If the younger age group was less affected by the cold weather, the actual benefits from the less stressful winters would be less obvious.

For ischemic heart disease and pneumonia, the relative effects of cold weather to hot weather have been stable in the period under studied. It appeared that the effect of global warming may be evolving very slowly. In a relatively cold region like Korea, people were more sensitive to extreme hot weather as partly explained by acclimatization [27]. On the contrary, Hong Kong has a relatively warm climate. Indoor areas and transportations are mostly equipped with air-conditioning for cooling rather than warming. Therefore, people are less sensitive to hot weather than cold weather. The effect of global warming would tend to be milder. Our finding, which showed insignificant trend in the EMWS Index for ischemic heart disease, was consistent with our previous finding on the Excess Hospitalization in Winter vs. Summer Index for the same cause. Our previous finding showed that there was an absence of trend in excess winter hospitalization in the recent decade among those aged 65–84, and an increasing trend was observed for those aged 85 and above [36]. Since mortality involves a complex process, an increase in hospitalization may not necessarily be reflected in an increase in mortality. For example, it has been noted that improvement in technology and health services played a role in the time-varying nature of the relationship between temperature and mortality [27]. While the literature showed that circulatory or respiratory diseases were more sensitive to temperature or atmospheric pressure [1,6,7,35], mortality from cardiovascular diseases or pneumonia in the hot cities in the United States was not affected by either hot or cold temperatures [5]. Since Hong Kong is a relatively warm city, it may partly explain the insignificant trend in the EMWS Index for ischemic heart disease and pneumonia in Hong Kong. Global warming appeared not to have an observable effect.

Meanwhile, the declining trend in excess winter mortality from other causes of death were not fully explained by extreme weather variables, suggesting the presence of factors other than climate changes. This was reasonable because of the heterogeneous causes of deaths included in this category. Actually, the EMWS Index of other causes was substantially smaller than the indices for the circulatory or respiratory diseases which had more prominent excess winter mortality.

There were results that were different from our expectation. An increase in the EMWS Index was associated an increase in the number of Very Hot Days (for chronic lower respiratory diseases); as well as increase in the number of very humid days and large daily temperature fluctuations (for pneumonia). The findings were unlikely a result of multicollinearity, because the largest VIF in all the models was just 2.45, far below the threshold of multicollinearity. It was uncertain if it was partly due to the reduced heat mortality resulted from issue of Very Hot Weather Warnings which was available since 2000 [43]. Alternatively, it might be related to human acclimatization [27]. When the elderly population was exposed to a prolonged period of stressful summer, reflected by the larger number of days with the extreme weather conditions, the survivors might be more sensitive to the cold weather in winter even if the winter is not particularly stressful. There may be other confounding factors like air pollution or influenza epidemics. Extreme summer weather may also trigger some diseases that had chronic lower respiratory diseases or pneumonia as complications, and with delayed death beyond summer. Further investigations were needed.

There are strengths and limitations in this study. The mortality data from the Census and Statistics Department captured all deaths in Hong Kong as it is mandatory to report deaths to the government. Our study utilized all data available at the time of analysis, spanning a time-series of 35 years, such that reliable trends could be established. While there were studies investigating the adverse effect of cold weather on mortality among the older population living at the sub-tropical region [13,14], our study was the first study to quantify the relative effect of winter weather compared to summer weather on the mortality, via the use of the EMWS Index. Nevertheless, owing to the observational nature of the study, the findings only show correlation instead of causation. While this study included extreme weather variables, there might be potential confounding factors, like air pollution or influenza epidemics, which this study had not accounted for. In this study, the thresholds of extreme weather were mainly adopted from official observatory or from literature. However, there were still some thresholds that were set arbitrarily, such as those for relative humidity and atmospheric pressure. While we used extreme values to identify the stressful conditions, changes in the weather conditions may be a factor. For example, pressure trend, which was defined as the pressure today against pressure yesterday, was found to affect mortality during heat waves [44]. There may be factors other than meteorological factors, such as individual’s medical history, human acclimatization, improvement in health services and socioeconomic status, that might affect the trend in the mortality [27,45,46]. However, we were unable to control them in the current study.

## Supporting Information

S1 Appendix International Classification of Diseases (ICD) codes of selected causes of death. (TIF)
