# Supplementary figures and images for: The Trends in Excess Mortality in Winter vs. Summer in a Sub-Tropical City and Its Association with Extreme Climate Conditions
Source: PLoS One. 2015 May 20;10(5):e0126774. doi: 10.1371/journal.pone.0126774 (PMC4439064; doi:10.1371/journal.pone.0126774)

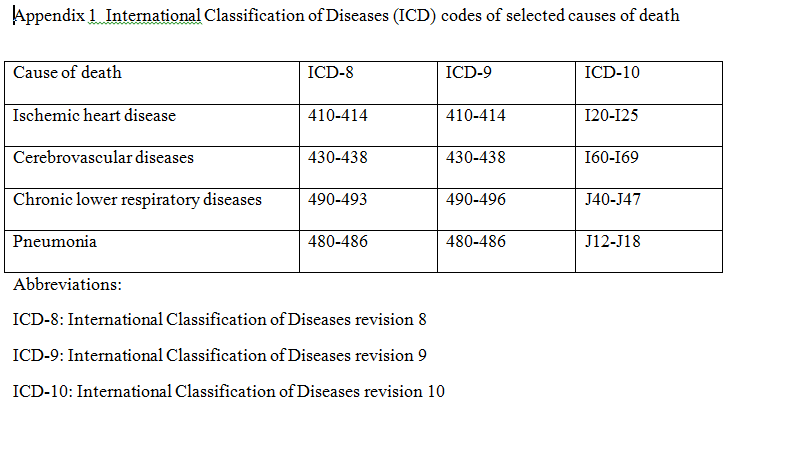

Supplement: S1 Appendix — (TIF) [file pone.0126774.s001.tif]
